# Transcriptome Analysis Reveals the Different Response to Toxic Stress in Rootstock Grafted and Non-Grafted Cucumber Seedlings

**DOI:** 10.3390/ijms21030774

**Published:** 2020-01-24

**Authors:** Xuemei Xiao, Jian Lv, Jianming Xie, Zhi Feng, Ning Ma, Ju Li, Jihua Yu, Alejandro Calderón-Urrea

**Affiliations:** 1 College of Horticulture, Gansu Agricultural University, Lanzhou 730070, China; xiaoxm@gsau.edu.cn (X.X.); lvjian@gsau.edu.cn (J.L.); fengz@gsau.edu.cn (Z.F.); Maning96@139.com (N.M.);; 2 Gansu Provincial Key Laboratory of Aridland Crop Science, Gansu Agricultural University, Lanzhou 730070, China; 3 College of Plant Protection, Gansu Agricultural University, Lanzhou 730070, China; calalea@csufresno.edu; 4 Department of Biology, College of Science and Mathematics, California State University, Fresno, CA 97340, USA

**Keywords:** grafting, cucumber, autotoxic stress, cinnamic acid, transcriptome, phtotosyntesis

## Abstract

Autotoxicity of root exudates is one of the main reasons for consecutive monoculture problem (CMP) in cucumber under greenhouse cultivation. Rootstock grafting may improve the tolerance of cucumber plants to autotoxic stress. To verify the enhanced tolerance to autotoxic stress and illuminate relevant molecular mechanism, a transcriptomic comparative analysis was performed between rootstock grafted (RG) and non-grafted (NG) cucumber plants by a simulation of exogenous cinnamic acid (CA). The present study confirmed that relatively stable plant growth, biomass accumulation, chlorophyll content, and photosynthesis was observed in RG than NG under CA stress. We identified 3647 and 2691 differentially expressed genes (DEGs) in NG and RG cucumber plants when compared to respective control, and gene expression patterns of RNA-seq was confirmed by qRT-PCR. Functional annotations revealed that DEGs response to CA stress were enriched in pathways of plant hormone signal transduction, MAPK signaling pathway, phenylalanine metabolism, and plant-pathogen interaction. Interestingly, the significantly enriched pathway of photosynthesis-related, carbon and nitrogen metabolism only identified in NG, and most of DEGs were down-regulated. However, most of photosynthesis, Calvin cycle, glycolysis, TCA cycle, and nitrogen metabolism-related DEGs exhibited not or slightly down-regulated in RG. In addition, several stress-related transcription factor families of AP2/ERF, bHLH, bZIP, MYB. and NAC were uniquely triggered in the grafted cucumbers. Overall, the results of this study suggest that rootstock grafting improve the tolerance of cucumber plants to autotoxic stress by mediating down-regulation of photosynthesis, carbon, and nitrogen metabolism-related DEGs and activating the function of stress-related transcription factor. The transcriptome dataset provides an extensive sequence resource for further studies of autotoxic mechanism at molecular level.

## 1. Introduction

Consecutive monoculture problem (CMP) happens when one plant is cultivated in the same field year after year, which results in growth decline, crop yield reduction, quality degradation, and disease susceptibility. Previous studies have indicated that the causes of the CMP include soil physicochemical properties deterioration, microbial community structure imbalance, and autotoxicity of root exudates [1,2,3]. Cucumber (*Cucumis sativus* L.) suffers from a severe consecutive monoculture problem especially under greenhouse cultivation, and the main cause for this CMP in cucumber is the secretion of autotoxins. Yu and Matsui [4] identified ten kinds of autotoxins, including cinnamic acid (CA), from cucumber root exudates. Furthermore, it was subsequently demonstrated that CA inhibited plant growth and photosynthesis of cucumber. Interestingly, the incidence of Fusarium wilt caused by *Fusarium oxysporum* f. sp. *cucumerinum* of cucumber increased after cinnamic acid treatment [5,6]. Thus, finding a solution to CMP caused by cinnamic acid in cucumber is of paramount importance.

Extensive research has been conducted to alleviate CMP in cucumber and some of these include using different cultivation patterns, using beneficial microbe detoxification, and grafting. For example, rotation with garlic, leafy vegetables or *Volvariella volvacea* improved the composition of soil microbes and decreased Fusarium wilt of cucumber [7,8]. Similarly, intercropping with garlic and green garlic alleviated CMP in cucumber by altering soil microbial communities [9,10]. Some beneficial microorganisms were applied to degrade cinnamic acid thereby mitigating the allelopathic stress; these beneficial microorganisms include *Trichoderma harzianum* SQR-T037 [11], *Phomopsis liquidambari* [12], and *Stenotrophomonas* sp. TRMK2 [13]. Finally, grafting has been reported as an effective strategy to improve the growth and increase plant tolerance to abiotic stress. It was reported that grafted-root watermelon secreted less cinnamic acid but more chlorogenic acid and caffeic acid than the own-root watermelon and it enhanced resistance to *Fusarium oxysporum* f. sp. *niveum* [14]. Likewise, grafting improved plant growth and root antioxidant capacity of eggplant under cinnamic acid [15]. In cucumber, only one report suggested that cucumber, but not its rootstock figleaf gourd, suffered from oxidative stress upon exposure to cinnamic acid [16]. In the current study, we hypothesized that rootstock-grafted (RG) and non-grafted (NG) cucumber plants showed different response to cinnamic acid stress.

Recently, transcriptomic studies have been applied as a powerful tool to better understand the molecular mechanisms involved with resistance to abiotic stress by grafting, such as low temperature [17] and drought stress [18]. A number of differentially expressed genes (DEGs) related to rootstock grafted watermelon regulated chilling tolerance were identified, which are involved in induction of protein processing, plant–pathogen interaction, the spliceosome, and suppression of photosynthesis [17]. It was also discovered based on transcriptomic analysis that grafted tomato could better adapt to drought stress by regulating ABA signal transduction and stomatal aperture [18]. These studies suggested grafting could enhance plant tolerance to abiotic stress by mediating gene expression patterns and metabolic pathways. However, the overall molecular mechanism by which grafting alters cinnamic acid tolerance remains largely unclear.

In the present study, we demonstrate that the rootstock-grafted cucumber (RG) seedlings are more tolerant to cinnamic acid (CA) stress than non-grafted (NG) cucumber seedlings. Furthermore, the different gene expression between RG and NG cucumber seedlings under CA stress was conducted using an Illumina sequencing based transcriptomic approach. We found more genes were differentially expressed in NG than RG seedlings, and these genes were enriched in more than one hundred pathways involved in photosynthesis, signal transduction, secondary metabolism, and plant–pathogen interaction. Therefore, it appears that CA triggers a very complex response in susceptible cucumber seedlings.

## 2. Results

### 2.1. Plant Morphology and Biomass

To identify the different tolerance to cinnamic acid (CA), stress of rootstock grafted (RG), and non-grafted (NG) cucumber seedlings, the plant parameters were evaluated after 7 days of treatment. Plant height, stem diameter, and leaf area of NG seedlings were significantly decreased compared with control seedlings, but no significant differences in RG seedlings were observed (Table 1). Compared with 0 mM CA, the 0.5 mM CA treatment significantly inhibited the shoot fresh (20.3%) and dry weight (42.8%) of NG seedlings. Only a slight decrease in the plant biomass of RG seedlings was observed under CA stress. These results indicate that rootstock grafting can improve the tolerance to CA stress in cucumber.

### 2.2. Chlorophyll Contents and Gas Exchange Parameters

**Figure 1:**
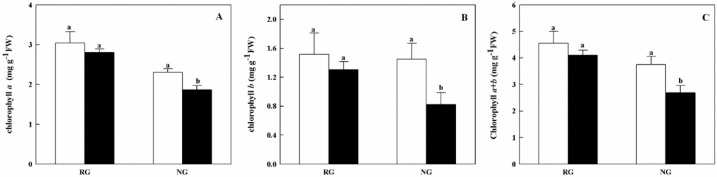
Effects of cinnamic acid (CA) on the chlorophyll *a* (**A**), *b* (**B**) and *a + b* (**C**) contents of rootstock grafted (RG) and non-grafted (NG) cucumber seedlings. Leaves samples were taken 7 d after exposed to 0 mM (white bars) and 0.5 mM CA (dark bars). Each histogram represents a mean value of three biological replicates, and the vertical bars indicate SD (*n* = 3). Bars sharing the same letters are not significantly different at the 5% level according to the least significant difference test (LSD).

To further understand physiological variation in RG and NG cucumber seedlings, the photosynthetic capacity was assessed. As shown in Figure 1, no significant difference of total chlorophyll contents was found in RG seedlings between 0 mM and 0.5 mM CA treatments. For NG seedlings, the Chlorophyll a, b and a + b contents were significantly decreased by 22.5%, 50.9%, and 34.3% under CA stress, respectively.

**Figure 2:**
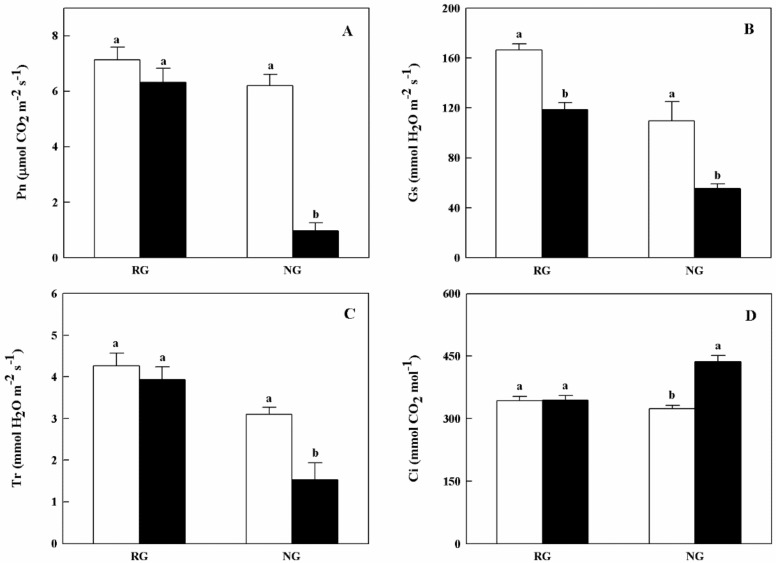
Effects of cinnamic acid (CA) on the gas exchange parameters of rootstock grafted (RG) and non-grafted (NG) cucumber seedlings 7 days after exposed to 0 mM (white bars) and 0.5 mM CA (dark bars). Each histogram represents a mean value of three biological replicates, and the vertical bars indicate SD (*n* = 6). Bars sharing the same letters are not significantly different at the 5% level according to the least significant difference test (LSD). (**A**): Pn, net photosynthetic rate; (**B**): Gs, stomatal conductance; (**C**): Tr, transpiration rate; Ci, (**D**): intercellular CO_2_ concentration.

Gas exchange parameters of RG and NG cucumber seedlings showed different response to CA treatment (Figure 2). Net photosynthetic rate (Pn) and transpiration rate (Tr) of NG seedlings were apparently reduced when treated with 0.5 mM CA. As for stomatal conductance (Gs), both RG and NG seedlings exhibited significantly decreased under CA stress. The inhibition rate of Gs in NG seedlings was nearly 2.0-fold higher than that of RG seedlings (Figure 2B). On the contrary, intercellular CO_2_ concentration (Ci) was significantly increased in NG seedlings under 0.5 mM CA treatment (Figure 2D).

### 2.3. Summary Analysis of mRNA Sequencing

Twelve cDNA libraries were established from RG and NG cucumber seedlings under 0 mM cinnamic acid (CK) or 0.5 mM cinnamic acid (CA) conditions for RNA-seq analysis. A total of 165.33, 144.58, 153.63, and 153.83 million raw reads were identified in the RG-CK, RG-CA, NG-CK, and NG-CA samples, respectively. After the adaptors, poly-N, and low quality reads filtering, 160.83 (97.28%), 140.81 (97.39%), 149.91(97.58%), and 150.73(97.98%) million clean paired-end reads of 125–150 bp in length were obtained for further analysis (Table 2).

Average of 93.76% of the clean reads across all the samples were mapped to the cucumber reference genome using Hisat2 [19], most of which were uniquely-mapped (91.96%). There were 17424, 17490, 17533, and 17503 expressed genes in the RG-CK, RG-CA, NG-CK, and NG-CA samples, respectively, accounting for 74.95%, 75.23%, 75.42%, and 75.29% of previous annotated genes in cucumber [20]. More than 90% of cDNA sequencing quality was ≥ 30 (Q30), suggesting that sequencing data are of high quality and reliable for subsequent bioinformatics analysis.

### 2.4. Identification of Differentially Expressed Genes (DEGs)

**Figure 3:**
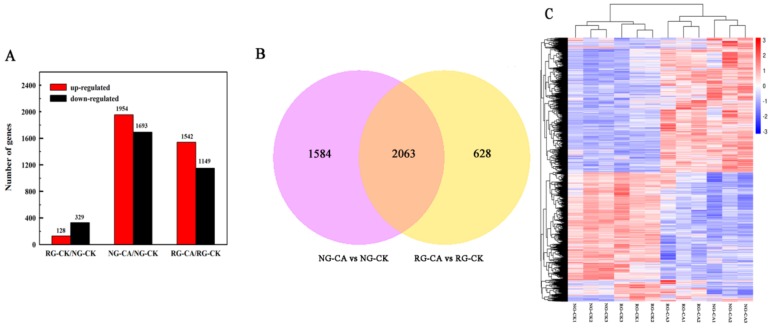
Number of significantly different differentially expressed genes (DEGs) among the libraries. (**A**). Number of up- and down-regulated DEGs among RG and NG cucumber seedlings exposure to 0 mM (CK) and 0.5 mM cinnamic acid treatment (CA) libraries. (**B**). Venn diagram of DEGs under the control and CA stress conditions. Significant difference of DEGs was expressed at the two criterions of |log_2_FoldChange| > 1 and Padj < 0.05. (**C**). Cluster analysis of DEGs among 12 samples using hierarchical clustering method. Expression of the same gene among different samples was shown in the horizontal direction. Red color represents high expression and blue color represents low expression.

A total of 457 DEGs were observed in RG compared to NG seedlings under control (Table S2, Figure 3A). Of these, 128 were up-regulated and 329 were down-regulated. In total, 3647 genes were differentially expressed in NG seedlings treated with 0.5 mM CA, with 1954 up- and 1693 down-regulated (Table S3, Figure 3A). Only 2691 genes (1542 up- and 1149 down-regulated) were differentially expressed in RG cucumber seedlings (Table S4, Figure 3A). Furthermore, 2063 DEGs were induced by CA stress in both RG and NG seedlings (Table S5, Figure 3B). A total of1584 and 628 cinnamic acid-responsive genes were uniquely observed in NG and RG, respectively. The sequencing results suggested that the more abundant genes, and corresponding molecular regulatory processes of, NG than that of RG were regulated by CA stress. The differentially expressed genes (DEGs) were identified using DESeq2 following the criterion of adjusted *p*-value < 0.05 and |log_2_ Fold Change| ≥ 1.

A hierarchical clustering analysis was performed on DEGs to explore the relationship between samples (Figure 3C). Two large clusters were established from 12 samples, with one containing RG and NG samples under control conditions and another cluster containing samples treated with 0.5 mM CA. Interestingly, the levels of gene expression between the two clusters showed opposite patterns, showing a higher gene expression in the group treated with CA. Therefore the results suggest that the effect of CA stress on gene expression of cucumber seedlings is stronger than grafting.

### 2.5. Functional Classifications of DEGs Response to Grafting

Gene Ontology (GO) term and KEGG pathway analysis was conducted to examine the potential gene function and metabolism pathway of DEGs. As shown in Table S6, a total of 283 assigned GO terms from 457 DEGs between RG and NG seedlings was obtained. Among these, 129, 22, and 132 GO terms were categorized into biological process, cellular component, and molecular function categories, respectively. GO terms associated with transport (GO:0008272, sulfate transport; GO:0072348, sulfur compound transport; and GO:0015698, inorganic anion transport) and metabolic process (GO:0044262, cellular carbohydrate metabolic process; GO:0006073, cellular glucan metabolic process; and GO:0009308, amine metabolic process) were enriched in biological process classification. As for cellular component, the terms apoplast (GO:0048046), extracellular region (GO:0005576), cell wall (GO:0005618), and external encapsulating structure (GO:0030312) were significantly enriched. Moreover, DEGs involved in transcription factor activity (GO:0001071 and GO:0003700), transmembrane transporter activity (GO:0015103, GO:0015116, and GO:1901682), and hydrolase activity (GO:0016798 and GO:0004553) were dominant in molecular function classification (Figure S1).

A total of 82 DEGs responses to grafting were assigned to 55 KEGG pathways involved in metabolic pathways or signal transduction pathway (Table S7). The top 20 of KEGG pathways were shown in Figure S2. Among these KEGG pathways, glutathione metabolism (csv00480), and phenylpropanoid biosynthesis (csv00940) were significantly enriched.

### 2.6. Functional Classifications of DEGs Response to CA Stress

**Figure 4:**
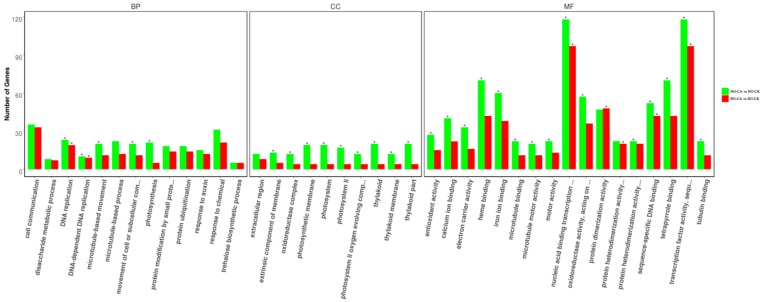
Gene ontology functional classification and enrichment analysis of DEGs in rootstock grafted (RG) and non-grafted (NG) libraries after cinnamic acid stress compared to their respective control. The asterisk represents significant enrichment of GO terms in RG-CA vs RG-CK and NG-CA vs NG-CK (Padj < 0.05). The x-axis represents GO classifications, y-axis represents the amount of DEGs in each classification.

To reveal the similarities and differences in CA stress-induced transcriptomes between RG and NG cucumber seedlings, separate DEGs were determined for GO classification and enrichment. A total of 3241 and 2357 DEGs in NG and RG cucumber seedlings between control and cinnamic acid stress were categorized into 744 and 695 functional groups using GO classifications, respectively (Table S8). Top 10 at least of GO terms categorized into biological process, cellular component, and molecular function, are shown in Figure 4. In the biological process category, DNA-dependent DNA replication (GO:0006261), DNA replication (GO:0006260), photosynthesis (GO:0015979), movement of cell or subcellular component (GO:0006928), and microtubule-based movement (GO:0007018) were significantly enriched in NG seedlings while only DNA-dependent DNA replication and DNA replication were significantly enriched in RG seedlings. Among the cellular components category, GO terms related to photosystem (GO:0009523, GO:0009579, GO:0044436, GO:0009521, GO:0009654, GO:0034357, and GO:0042651) and oxidoreductase (GO:1990204) were significantly enriched in NG seedlings. For the molecular function category, transcription factor activity and binding were the main groups in both NG and RG seedlings.

**Figure 5:**
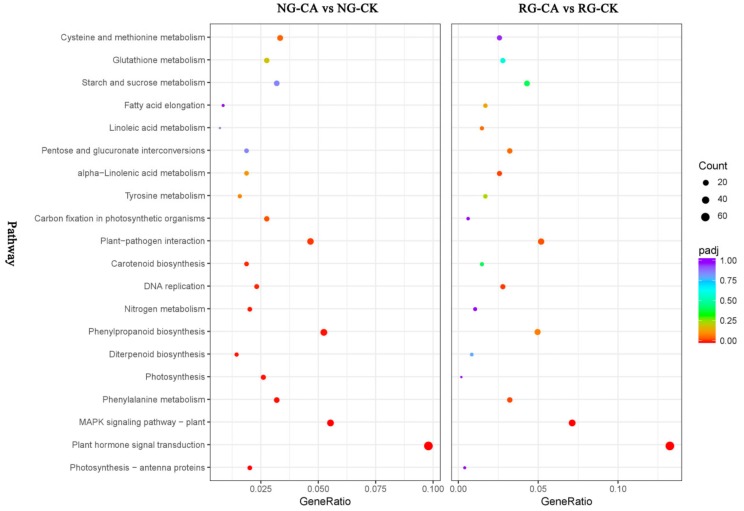
Top 20 of KEGG pathway maps of DEGs in RG and NG libraries after cinnamic acid stress compared to their respective control. The x-axis represents GeneRatio, which is the ratio of DEGs enriched to specific KEGG pathways to DEGs enriched to all KEGG pathways. The y-axis represents the KEGG pathways. The color closer to red of spot represents a higher significance of DEGs. Bigger size of spot represents more DEGs enriched.

Pathway enrichment analysis was performed to further understand the biological functions of DEGs. With transcriptome sequencing, 679 and 457 DEGs were assigned to 108 and 103 KEGG pathways in the NG and RG cucumber seedlings under CA stress compared to their respective control (Table S9). In NG seedlings, 344 up- and 335 down-regulated DEGs were enriched in 100 and 93 KEGG pathways, and in RG seedlings, 255 up- and 202 down-regulated DEGs were assigned to 88 and 74 KEGG pathways. The top 20 pathways with highest enrichment levels were identified in both NG and RG seedlings (Figure 5). Among these KEGG pathways, plant hormone signal transduction (csv04075), MAPK signaling pathway—plant (csv04016), phenylalanine metabolism (csv00360), plant–pathogen interaction (csv04626) and DNA replication (csv03030) were significantly enriched in NG and RG. However, the top three pathways in enrichment degree under NG and RG were different. In NG seedlings, the first three pathways were “photosynthesis—antenna proteins”, “plant hormone signal transduction” and “MAPK signaling pathway—plant”, while in RG seedlings were “plant hormone signal transduction”, “MAPK signaling pathway—plant”, and “DNA replication”. In addition, the most DEGs induced by CA stress enriched the top five pathways were plant hormone signal transduction (35 up- and 32 down-regulated DEGs), MAPK signaling pathway –plant (29 up- and 9 down-regulated DEGs), phenylpropanoid biosynthesis (29 up- and 7 down-regulated DEGs), plant–pathogen interaction (26 up- and 6 down-regulated DEGs), and carbon metabolism (20 up- and 28 down-regulated DEGs) in NG cucumber seedlings. As for RG seedlings, except for starch and sucrose metabolism (14 up- and 6 down-regulated DEGs), other top four pathways were the same as in NG. Interestingly, the count of DEGs in the same pathway was more in NG than RG seedlings. Furthermore, the significantly enriched pathways of photosynthesis—antenna proteins (csv00196), photosynthesis (csv00195), carbon fixation in photosynthetic organisms (csv00710), and nitrogen metabolism (csv00910) were only identified in NG cucumber seedlings, and most of DEGs was down-regulated. This suggested that the effect of CA stress treatment on photosynthesis and nitrogen metabolism of non-grafted cucumber seedlings was stronger than that of rootstock grafted cucumber seedlings.

### 2.7. DEGs Involved in Photosynthesis and Carbon Metabolism

To further understand how the photosynthesis and carbon metabolism-related genes in NG and RG cucumber plants response to CA stress, we extracted the DEGs involved in chlorophyll metabolism, photosynthesis—antenna proteins, photosynthesis, carbon fixation in photosynthetic organisms, carbon and nitrogen metabolism (Table S10). In chlorophyll metabolism, 11 DEGs were down-regulated in NG, and 4 of them were down-regulated in RG. The four DEGs were used to encode uroporphyrinogen decarboxylase, porphobilinogen deaminase, magnesium chelatase subunit I, and uroporphyrinogen decarboxylase, which showed lesser downregulation in RG than NG cucumber. In photosynthesis—antenna proteins and photosynthesis, all of 14 DEGs were used to encode Chlorophyll a-b binding protein, and were down-regulated both in NG and RG cucumber plants response to CA stress. Only two differentially affected genes ‘Csa1G445860′ and ‘Csa6G522690′ of them were found in RG, and the rate of down-regulation was lower than NG. In carbon fixation in photosynthetic organisms, 6 up-regulated and 13 down-regulated DEGs were exhibited in NG cucumber plants, but only 3 DEGs, ‘Csa3G150740′, ‘Csa5G577360′, and ‘Csa5G611700′ which encoded pyruvatephosphate dikinase, phosphoenolpyruvate carboxylase, and ribose-5-phosphate isomerase, were significantly up-regulated in RG cucumber plants. In carbon metabolism, 20 DEGs were up-regulated in NG and 10 DEGs were up-regulated in RG under CA stress, and the rate of up-regulation was lower in RG than NG except ‘Csa6G448740′. Two DEGs of ‘Csa3G904100′ and ‘Csa6G448730′ encoded ribose-phosphate pyrophosphokinase and threonine dehydratase were only observed in RG cucumber plants. Furthermore, 28 and 5 down-regulated DEGs were found in NG and RG at an equal level. Surprisingly, only one DEGs, ‘Csa1G615730′ encoded hexokinase 6, was identified in RG cucumber seedling. In nitrogen metabolism, 6 up-regulated and 8 down-regulated genes in NG, while one up-regulated and four down-regulated genes in RG cucumber plants, were differentially expressed when exposed to CA stress. Interestingly, four genes encoded carbonic anhydrase family protein were found in RG cucumber, and one of them was a novel gene.

### 2.8. Transcription Factors Encoded by DEGs

Among the DEGs of NG, 334 transcription factors were identified and classified into 46 TF families (Table S11), including AP2-EREBP (50), bHLH (18), C2H2 (10), HSF (10), NAC (20), MYB (34), WRKY (23), and C2C2-related (19) families. As shown in Supplementary Table S12, 276 transcription factors were identified in the DEGs of RG, including AP2-EREBP (42), bHLH (18), bZIP (12), HSF (7), MYB (25), NAC (21), C2C2 (17), and WRKY (21) families. Among the transcription factors responding to CA stress in NG and RG, 217 transcription factors were expressed in both NG and RG. In all 59 transcription factors were uniquely regulated in RG, including the AP2-EREBP (7), bHLH (5), MYB (5) and NAC (4) families.

### 2.9. Validation of mRNA-Seq by qRT-PCR

**Figure 6:**
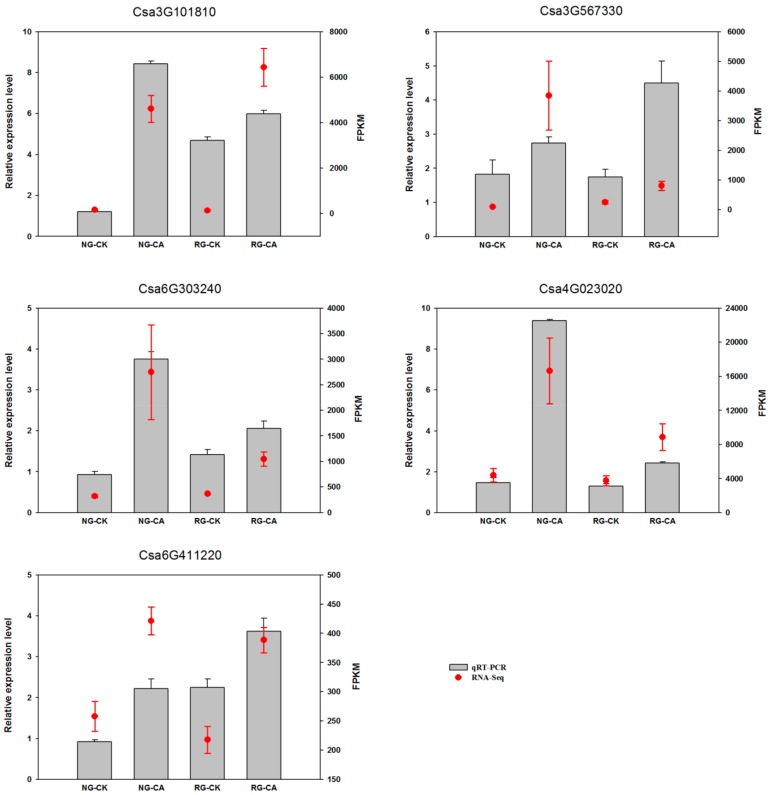
Validation of expression patterns of 5 randomly-selected DEGs by qRT-PCR. The x-axis indicates the four samples. NG: non-grafted cucumber; RG: rootstock grafted cucumber; CK: no treatment; CA: 0.5 mM cinnamic acid treatment. The left y-axis indicated relative expression level of qRT-PCR and the right y-axis indicates normalized expression level (FPKM) of RNA sequencing. Error bars represent standard error of mean.

Transcription factors can regulate expression of downstream genes and play a vital role in plant resistance to various biotic and abiotic stresses. To verify the reliability of RNA-Seq data, we randomly selected five transcription factor genes (Csa3G101810, Csa3G567330, Csa6G303240, Csa4G023020, Csa6G411220) for real-time PCR analysis (Figure 6). The qRT-PCR results showed that the expression patterns of the five genes were identical to those detected by transcriptome sequencing, which confirmed the reliability of RNA-seq data.

## 3. Discussion

Autotoxic stress is regarded as one of the many abiotic stresses observed in plants, and cinnamic acid (CA) is one of the stressors. The detrimental effects of CA on growth has been demonstrated in several plants, including cucumber [6], tomato [21], and watermelon [22]. Grafting is widely used in vegetable cultivation due to its resistant or tolerant to biotic or abiotic stress based on the well-developed root of the rootstock [23,24]. The aim of the present study was to verify the moderating effect of grafting on autotoxic stress of cucumber induced by CA, and to shed light on the potential molecular mechanisms involved using a transcriptome approach.

### 3.1. Overall Analysis of Gene Expression Based on RNA-seq

The RNA-Seq, quantitative RNA expression analysis, can be used to accurately monitor gene expression in different tissues or conditions without sophisticated normalization of data sets [25]. In our study, the trancriptomic analysis showed relatively fewer DEGs related to CA stress were obtained in RG (2691 DEGs) than that in NG (3647 DEGs) cucumber seedlings. We speculate that rootstock grafting improved the resistance of cucumber seedling to CA stress due to lower sensitivity. Similar results were also obtained by [17], where weaker response to chilling stress in rootstock grafted than self-grafted watermelon seedlings. In addition, it was found that less of DEGs in RG libraries (42.7%) were down-regulated after exposure to CA treatment compared to NG (46.4%), which indicated that rootstock grafting could keep gene expression stable under adverse condition.

### 3.2. Discrepancy of Morphology and Photosynthesis between NG and RG Cucumber Seedlings

It is well known that *Cucurbita ficifolia* as rootstock is conducive to growth of scion even under stress conditions. Previous studies found higher dry weight when cucumber grafted onto figleaf gourd than self-grafted plants under salt stress [26]. Similarly, grafting onto figleaf gourd alleviated the declined dry weight of cucumber caused by chilling [27,28] reported stem girth, leaf area, and dry weight were significantly enhanced in figleaf gourd rootstock-grafted than that of non-grafted cucumber plants during winter under unheated greenhouse, subsequently accompanied by 30% yield increase. In the present study, the result also showed that decrease of plant height, stem diameter, leaf area and dry matter contents of cucumber under CA stress was adjusted by grafting onto figleaf gourd (Table 1).

Chlorophylls, as the core component of pigment–protein complexes, play a key role in photosynthesis. Previous studies reported that reduced chlorophyll content appeared in allelochemical-treated plants [5,29]. Similar phenomenon also observed in non-grafted cucumber when exposure to CA treatment in this study. However, no significant difference of chlorophyll *a*, *b* and *a + b* was found in rootstock grafted cucumber compared to control. The result indicated grafting onto figleaf gourd regulated the reduction of Chl accumulation in cucumber leaves, which are consistent with the results obtained by [30]. Furthermore, the transcriptome data showed that the expression levels of Chl synthesis-related genes were significantly down-regulated in NG while they were not or slightly down-regulated in RG cucumber when compared to respective control (Table S10). The Mg-chelatase catalyzes the insertion of Mg^2+^ into the protoporphyrin IX which is the first step in the chlorophyll biosynthesis pathway [31]. The two genes (Csa4G165920, Csa6G518380) encoded Mg-chelatase was 1.26 and 1.84 fold down-regulated in NG while one gene (Csa6G518380) of them was 1.10 fold down-regulated in RG cucumber. Another key enzyme is chlorophyll synthase, which catalyzes the last step of chlorophyll biosynthesis. We found the down-regulation gene encoded chlorophyll synthase was observed only in NG cucumber seedlings under CA stress. The results suggest that grafting improve the tolerance of cucumber to CA stress likely by attenuated inhibition of Chl synthesis.

**Figure 7:**
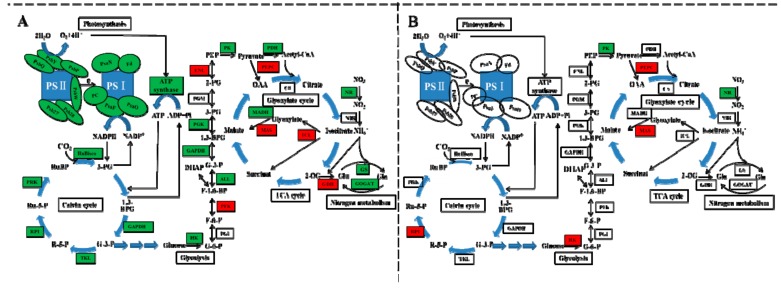
Schematic representation of carbon-nitrogen metabolism changes in non-grafted cucumber (**A**) and rootstock grafted cucumber (**B**) exposed to CA stress. Photosynthesis, Calvin cycle, glycolysis, TCA cycle, glyoxylate cycle and nitrogen metabolism were regulated by grafting. Different color indicates level of gene expression with red representing up-regulation while green as down-regulation. The level of regulation was determined based on log_2_ fold change. The blue arrow represents the direction of substance metabolism in cyclic metabolic pathway. [Abbreviations for different subunits: PSII reaction centre core protein (PsbY, PsbW, Psb27, Psb28); subunit of oxygen evolving complex protein (PsbP); oxygen-evolving enhancer protein (PsbQ); PSI reaction center subunit N (PsaN); PSI reaction center subunit III (PsaF); sex-linked protein 9 (PsaO); Ferredoxin (Fd); Plastocyanin (PC); Abbreviations for enzymes: ribulose-1,5-bisphosphate carboxylase/oxygenase (RuBisco); glyceraldehyde 3-phosphate dehydrogenase (GAPDH); transketolase (TKL); ribose-5-phosphate isomerase (RPI); phosphoribulokinase (PRK); hexokinase (HK); phosphoglucose isomerase (PGI); phosphofructokinase (PFK); aldolase (ALL); phosphoglycerate kinase (PGK); phosphoglycerate mutase (PGM); enolase (ENL); pyruvate kinase (PK); pyruvate dehydrogenase (PDH); phosphoenolpyruvate carboxylase (PEPC); citrate synthase (CS); isocitrate lyase (ICL); malate synthase (MAS); malate dehydrogenase (MADH); glutamate dehydrogenase (GDH); glutamate synthase (GOGAT); glutamine synthetase (GS); nitrite reductase (NiR), nitrate reductase (NR); Abbreviations for metabolites: ribulose biphosphate (RuBP), 3-phosphoglycerate (3-PG), 1,3-bisphosphoglycerate (1,3-BPG); G-3-P, ribose 5-phosphate (R-5-P); ribulose5-phosphate (Ru-5-P); glucose 6-phosphate (G-6-P); fructose 6-phosphate (F-6-P); fructose 1,6-bisphosphate (F-1,6-BP); 2-phosphoglycerate (2-PG); phosphoenolpyruvate (PEP); oxaloacetate (OAA); 2-oxoglutarate (2-OG); glutamate (Glu); glutamine (Gln)].

Exogenous cinnamic acids at 0.25 mM significantly inhibited gas exchange parameters of cucumber seedlings, including net photosynthetic rate (Pn), intercellular CO_2_ concentration (Ci), leaf transpiration (Tr), and stomatal conductance (Gs) [5]. We also found that photosynthetic parameters of non-grafted cucumber declined under CA stress except Ci (Figure 2), possibly because of the conversion from stomatal to non-stomatal control under different concentrations of CA. However, after grafted onto figleaf gourd, photosynthetic parameters of cucumber leaf, with the exception of Gs, exhibited steady state. A similar report of grafting triggering stronger photosynthetic ability was found in citrus under abiotic stress [30]. All of 14 DEGs encoded chlorophyll a-b binding protein were down-regulated in NG while two of them were down-regulated in RG, and the rate of descent was lower in RG than NG (Table S10). Additionally, all of the down-regulated DEGs encoded pivotal proteins in photosynthesis containing Photosystem I reaction center subunit N, Photosystem II PsbY/Psb27/Psb28/PsbW, and ATP synthase, and they were only identified in NG response to CA (Figure 7). Thus, we suggested that the photosynthesis ability of RG was stronger than that of NG under CA stress benefiting from regulation of photosynthesis-related genes.

### 3.3. Carbon and Nitrogen Metabolism Analysis

Photosynthetic carbon fixation of cucumber, a C3 plant, uses the Calvin cycle to generate its primary product of 3-phosphoglycerate. Several enzymes participate in the carbon fixation reaction and other metabolic processes. The basic reaction is catalyzed by ribulose-l,5-bisphosphate carboxylase (RuBisco) using RuBP and CO_2_ as substrate. In the present study, DEG (Csa5G609710) encoded Ribulose bisphosphate carboxylase small chain was 1.18 fold down-regulated in NG under CA stress compared to control, but no difference was found in RG (Table S10, Figure 7). Similar results were obtained in DEGs encoding other enzymes of ribose 5-phosphate isomerase, glyceraldehyde 3-phosphate dehydrogenase, phosphoglycerate kinase, and fructose-1-6-bisphosphatase, which are related to leaf area expansion and fresh weight accumulation [32]. This could partially explain why leaf area and fresh weight of NG cucumber plant declined under CA treatment (Table 1). Surprisingly, Csa5G577360 encoded phosphoenolpyruvate carboxylase (PEPC) protein was up-regulated both in NG and RG cucumber seedlings, and up-regulated rate was higher in NG than RG. Bradbeer et al. [33] found a decline in RuBisco activity and an increase in PEPC activity in etiolated primary leaves of *Phaseolus*. Therefore, we conjecture that up of gene expression involved in PEPC might contribute to the decrease of chlorophyll contents in NG.

Glycolysis and tricarboxylic acid (TCA) cycle are two key metabolic pathways participating in carbohydrate metabolism to produce energy for ensuring the normal life activities. Previous studies indicated the carbohydrate metabolism was inhibited under abiotic stress due to the decrease of key enzymes involved in glycolysis and tricarboxylic acid cycle. The level of glyceraldehyde-3-phosphate dehydrogenase (GAPDH), triose phosphate isomerases, isocitrate dehydrogenase, and one malate dehydrogenase was down-regulated by salt stress through proteomic analysis [34]. In this study, three of DEGs (Csa3G252490, Csa4G664300, and Csa1G050250) encoded GAPDH were significantly down-regulated only in NG under CA stress (Table S10). Some other important genes encoding key enzymes involved in glycolysis (Csa6G449820, Csa3G359130, pyruvate kinase; Csa6G136020, phosphoglycerate kinase; Csa2G252020, fructose-bisphosphate aldolase) were also down regulated in NG response to CA. However, only two down-regulated gene, Csa6G449820 and Csa3G199630, were identified in RG (Table S10, Figure 7). The results suggest that the energy production of non-grafted cucumber suffers from stronger inhibition than root-grafted cucumber when exposed to CA stress. In addition, the glyoxylate cycle, a bypassed pathway of the TCA cycle was induced by CA stress, which was correlated with leaf senescence [35]. Two of DEGs (Csa1G050360, Csa2G420990) encoding malate synthase and isocitrate lyase, two key enzymes in the glyoxylate cycle, was up-regulated in NG, and one of that (Csa1G050360) was up-regulated in RG cucumber plants (Table S10) under CA stress. Similar phenomenon was also observed by Yuenyong et al. [36] where the isocitrate lyase-encoding gene (*OsICL*) in rice was highly induced by salt stress and then shift energy metabolism from the TCA cycle to the glyoxylate cycle.

A key enzyme connecting Carbon (C) and nitrogen (N) metabolism is phosphoenolpyruvate carboxylase, which is able to intermediate nitrogen assimilation by anaplerotic reaction in tricarboxylic acid cycle [37]. In the present study, the up-regulation of gene encoding PEPC implies conversion from C to N metabolism caused by CA stress. Furthermore, two of DGEs (Csa3G603580, Csa5G383350) encoding glutamate synthase (GOGAT) and nitrate reductase (NR), two key enzymes in N assimilation and uptake process, was dramatically down-regulated in NG response to CA (Table S10, Figure 7), which is consistent with those reports in other abiotic stress, such as salinity [38], drought [39], and extreme temperatures [40]. However, only Csa5G383350 was down-regulated in RG, and the down rate was 65% of that in NG. The result indicated grafting onto figleaf gourd could adjust the adverse effect of CA stress on growth and development of cucumber through maintaining relatively stable N metabolism.

### 3.4. Transcription Factor Analysis

Transcription factors play critical roles in plant resistance to various stresses by regulating expression of downstream genes. Several stress-related TFs of ethylene-responsive (AP2/ERF), basic helix-loop-helix (bHLH), Basic-leucine zipper (bZIP), MYB, and NAC were uniquely present in RG libraries under CA stress (Table S12).

The AP2/ERF family transcription factors have been shown function in the acquisition of stress tolerance by cross-talking with each other, and then regulate the developmental, physiological, and metabolic processes of plants [41]. Overexpression of *PsAP2* an AP2/ERF family in transgenic tobacco exhibited improved tolerance towards both abiotic and biotic stresses by activating *NtAOX1a* promoter [42]. In contrast, another AP2/ERF family gene *JcDREB2*-overexpressing rice displayed more sensitive to salt stress [43]. We found that 6 up-regulated and 1 down-regulated AP2/ERF family transcription factors genes in RG, which may be conductive to enhance CA tolerance in cucumber. Further study will be carried out to verify the function of AP2/ERF family transcription factors response to autotoxic stress by transgenic approach.

The MYB family is one of the largest TF families in plants, involving in diverse biological processes and abiotic stress responses in plants. Many R2R3-MYB proteins in various plants have been identified in response to multiple abiotic stresses by means of modulating secondary metabolism, plant hormone and signal conduction, or repressing protein phosphatase 2C (PP2C) genes [44,45,46,47]. The role of *VvMYB14* in the trans-activation of *VvSTS* genes has been confirmed by both silencing and over expression of *VvMYB14* in transgenic grapevine [44]. Stilbene synthase (STS) catalyzed the last step of phenylpropanoid pathway to produce stilbenes which play a role in response to biotic and abiotic stresses. Similar results were also obtained by [48], where lack of a complete subgroup of *R2R3-MYB* genes in *Arabidopsis thaliana* mutant seedling induced decrease of flavonol accumulation. As we known, the metabolism of cinnamic acid undergoes the phenylpropanoid pathway. In addition, the function of R2R3-MYB transcription factor regulating autotoxicity-related genes expression has been proved in the melon [49]. Therefore, we suggest that MYB domain transcription factor could transform the ability to defense autotoxic stress of cucumber by triggering the change of cinnamic acid metabolism.

## 4. Materials and Methods

### 4.1. Plant Materials and Cinnamic acid Treatments

Cucumber (*Cucumis sativus* cv. ‘Xinchun No.4′) was used as the scion and figleaf gourd (*Cucurbia ficifolia* Bouché) was used as the rootstock. Seeds of rootstock were sown into nursery filled with silica sand, and scion seeds were sown 3 days later. When the cotyledon of the cucumber fully expanded, a ‘top insertion grafting’ was performed as described by [50]. Non-grafted ‘Xinchun No. 4′ cucumber seedlings as control were planted 3 days later than the cucumber used as scion. When the graft union completely healed approximately 7 d after grafting, rootstock grafted (RG) and non-grafted (NG) seedlings were transferred to 1 L plastic container containing four plants, six containers for each treatment. The 1/2 nutrient solution formula developed by [51] was used as the base solution and renewed every 2 d. All plastic containers were put into growth chambers at 14/10 h photoperiod, 28/21 °C day/night temperatures, a relative humidity of 75%, and an illumination intensity of 30,000 lux.

After the full extension of the third true leaves, the rootstock grafted (RG) and non-grafted (NG) cucumber seedlings were treated with 0.5 mM cinnamic acid (CA). Eighteen plants were chosen for each treatment and were done in three biological replicates. The third leaf samples were collected at 8 h after exposure to CA treatment, frozen immediately in liquid nitrogen and stored at –80 °C until RNA isolation. Seven days later, plant growth and leaf photosynthesis were measured.

### 4.2. Plant Morphology and Biomass

Nine plants were randomly selected from each treatment to determine the growth index. Plant height was measured from the apical meristem to basal part of stem with a straightedge. Stem diameter was measured at 1 cm up from the cotyledon using a digital Vernier caliper. Leaf area of the fourth fully expanded leaves was measured using a scanner (EPSON Scan, Regent, Canada). To determine the plant biomass, the samples were washed in distilled water, dried with tissue paper and weighed as fresh weight. The dry weight was recorded after the shoot and root parts of plant were oven dried at 105 °C for 15 min and at 75 °C for 72 h.

### 4.3. Leaf Photosynthesis

The chlorophyll *a* and *b* contents were determined according to [52]. Namely, the fourth fully matured leaves from three plants was sampled, homogenized in 80% acetone and measured using a spectrophotometer (UV-1800; Shimadzu, Kyoto, Japan) at 663 and 665nm. The net photosynthetic rate (Pn), stomatal conductance (Gs), intercellular CO_2_ concentration (Ci), and transpiration rate (Tr) were measured using a portable photosynthesis system (CIRAS-2, PP-system, UK). The assimilatory chamber attached to one leaf was controlled to maintain the photosynthetic photon flux density, leaf temperature, relative humidity and CO_2_ concentration at 1000 μmol m^−2^ s^−1^, 25 °C, 75% and 380 μmol mol^−1^, respectively.

### 4.4. RNA-Seq Library Construction and Sequencing

RNA-Seq library construction and sequencing were conducted by Beijing Novogene Technology Co. Ltd. (Beijing, China). A total amount of 3 μg RNA per sample was used as input material for the RNA sample preparations. Sequencing libraries were generated using NEBNext^®^ UltraTM RNA Library Prep Kit for Illumina^®^ (NEB, USA) following the manufacturer’s recommendations. Briefly, mRNA was purified from total RNA using poly-T oligo-attached magnetic beads. Fragmentation was carried out using divalent cations under elevated temperature in NEBNext First Strand Synthesis Reaction Buffer (5X). First strand cDNA was synthesized using random hexamer primer and M-MuLV Reverse Transcriptase (RNase H-), followed by second strand cDNA synthesis using DNA Polymerase I and RNase H. In order to select cDNA fragments of preferentially 250~300 bp in length, the library fragments were purified with AMPure XP system (Beckman Coulter, Beverly, USA). Then PCR was performed with Phusion High-fidelity DNA Polymerase and universal PCR primers. Finally, PCR products were purified (AMPure XP system) and library quality was assessed on the Agilent Bioanalyzer 2100 system. The prepared library was sequenced on an Illumina Hiseq 6000 platform (Illumina, San Diego, CA, USA) to generate 125 bp/150 bp paired-end reads.

### 4.5. Transcriptomic Analysis

Raw data (raw reads) of fastq format were firstly processed through in-house Perl scripts. In this step, clean data (clean reads) were obtained by removing reads containing adapter, ploy-N, and low quality reads. Clean reads from individual libraries of each group were mapped to the cucumber genome database (http://cucurbitgenomics.org/organism/2) using Hisat2 v2.0.5 [19]. The unique mapped reads were selected for downstream analyses. The FPKM (fragments per kilobase of per millions fragments mapped) of each gene was calculated based on the length of the reads count mapped gene and considered as the most commonly used method for estimating gene expression levels. The formula of FPKM is as follows: FPKM = (10^6^*nf) / (L*N), where nf represents the number of fragments mapped to target gene, L represents the number of kilobase of the target gene, N represents the number of total fragments mapped to reference genome. Differential expression analysis of two treatments/groups (three biological replicates) was performed using the DESeq2 R package (1.16.1) [53]. DESeq2 provide statistical routines for determining differential expression in digital gene expression data using a model based on the negative binomial distribution. The resulting *p*-values were adjusted using the Benjamini and Hochberg’s approach for controlling the false discovery rate. Genes with an adjusted *p*-value < 0.05 and |log_2_ Fold Change| ≥ 1 found by DESeq2 were assigned as differentially expressed between two samples.

Gene Ontology (GO) and KEGG pathways enrichment analysis of differentially expressed genes was implemented by the cluster Profiler R package, in which gene length bias were corrected [54]. The corrected *p* value less than 0.05 were considered significantly enriched.

### 4.6. Validation of mRNA-Seq by Quantitative Real-Time PCR (qRT-PCR)

Five genes from four samples were randomly selected for qRT-PCR analysis to validate the mRNA-Seq data. 0.2 μg total RNA which was the same used for mRNA-sequencing, was used for first strand cDNA synthesis using the SuperScript^®^ III RT (Invitrogen, USA). The sequences of the primers used for qRT-PCR are listed in Supplementary Table S1. The qRT-PCR was performed on a StepOne TM Real-Time PCR System (Applied Biosystems, USA) with SYBR qRCR Mix (Invitrogen, USA) according to manufacturer’s instructions. The reaction system was consisted of 10 μL qRCR Mix, 1 μL upstream primer (10 μmol L^−1^), 1 μL downstream primer (10 μmol L^−1^), 2 μL cDNA template, and ddH_2_O added to the mixture for a total volume of 20 μL. The thermal cycling profile was 95 °C for 5 min, followed by 40 cycles of 95 °C for 10 s, 58 °C for 20 s, and a final extension of 20 s at 72 °C. The relative expression level normalized against *actin* (the internal standard gene) was expressed as 2^−ΔΔC^_T_ [55].

## 5. Conclusions

The present study confirmed that grafting onto rootstock (*Cucurbia ficifolia* Bouché) could alleviate the detrimental effect of CA stress on plant growth, biomass accumulation, chlorophyll content, and photosynthesis ability compared to non-grafted cucumber seedlings. In addition, on the basis of transcriptomic analysis, we found that most of the key DEGs related to photosynthesis, calvin cycle, glycolysis, TCA cycle and nitrogen metabolism response to CA stress were only present or higher down-regulated in NG cucumber plants (Figure 7). However, the gene encoding isocitrate lyase participating in glyoxylate cycle was up-regulated only in NG cucumber plants exposed to CA stress. Grafting enhances toxic tolerance of cucumber plants mainly by regulating photosynthesis, carbon and nitrogen metabolism. The transcription factors response to CA stress were also screened and analyzed. We identified several stress-related transcription factor families of AP2/ERF, bHLH, bZIP, MYB, and NAC uniquely triggered in the grafted cucumbers. This study provides a better understanding of the mechanism of grafting enhances cucumber tolerance to CA stress at the molecular level.

## Figures and Tables

**Table 1:** Growth parameters of rootstock grafted and non-grafted cucumber seedlings under cinnamic acid (CA) stress.

Treatment	Plant Height (cm)	Stem Diameter (mm)	Leaves Area (cm^2^)	Shoot		Root	
				Fresh Weight (g)	Dry Weight (g)	Fresh Weight (g)	Dry Weight (g)
RG-CK	14.67 ± 1.76 a	7.96 ± 0.13 a	432.81 ± 2.61 a	12.13 ± 0.09 a	1.11 ± 0.010 a	2.54 ± 0.23 a	0.13 ± 0.010 a
RG-CA	15.17 ± 1.04 a	7.86 ± 0.25 a	427.86 ± 10.44 a	12.02 ± 0.17 a	1.10 ± 0.021 a	2.12 ± 0.16 a	0.12 ± 0.012 a
NG-CK	14.17 ± 2.08 a	7.75 ± 0.46 a	420.68 ± 3.28 a	9.83 ± 0.20 a	0.73 ± 0.026 a	1.82 ± 0.06 a	0.07 ± 0.006 a
NG-CA	9.50 ± 0.50 b	7.37 ± 0.25 a	380.15 ± 5.72 b	8.16 ± 0.22 b	0.69 ± 0.017 a	1.45 ± 0.07 b	0.04 ± 0.006 b

The plant growth parameters were measured 7 days after exposed to 0 mM and 0.5 mM CA (*n* = 9). Means ± SD followed by the same letter are not significantly different at the 5% level according to the least significant difference test (LSD). RG-CK, rootstock grafted cucumber seedling under control; RG-CA, rootstock grafted cucumber seedling under CA stress; NG-CK, non-grafted cucumber seedling under control; NG-CA, non-grafted cucumber seedling under CA stress.

**Table 2:** Summary of transcriptome sequencing data.

Sample Name	Raw Reads	Clean Reads	Total Mapped Ratio (%)	Unique Mapped Ratio (%)	Q30 Percentage (%)	Expressed Genes	Expressed Genes Ratio (% of Annotated Genes)
RG-CK-1	60065280	58347004	93.64%	91.81%	89.84	17434	74.99
RG-CK-2	52976972	51443526	93.71%	91.92%	90.03	17386	74.78
RG-CK-3	52288760	51039256	94.32%	92.45%	90.83	17453	75.07
RG-CA-1	46296420	45246686	93.23%	91.53%	88.34	17629	75.83
RG-CA-2	49512360	48120914	93.17%	91.27%	89.82	17564	75.55
RG-CA-3	48778530	47442262	92.92%	90.96%	90.11	17279	74.32
NG-CK-1	50039680	48367266	93.51%	91.83%	89.96	17686	76.08
NG-CK-2	59498560	58473188	94.13%	92.32%	90.53	17484	75.21
NG-CK-3	44090680	43071056	94.1%	92.35%	90.56	17430	74.97
NG-CA-1	48323566	47469736	93.98%	92.28%	90.43	17467	75.13
NG-CA-2	54922480	53952812	94.08%	92.27%	90.81	17626	75.82
NG-CA-3	50581658	49314228	94.35%	92.58%	90.72	17417	74.92

Samples for mRNA-Seq derived from the fully expanded third leaves at 8 h after exposure to 0 mM and 0.5 mM CA. Q30: DNA sequencing quality ≥ 30. RG-CK, rootstock grafted cucumber seedling under control; RG-CA, rootstock grafted cucumber seedling under CA stress; NG-CK, non-grafted cucumber seedling under control; NG-CA, non-grafted cucumber seedling under CA stress.

## Supplementary Materials

Supplementary materials can be found at https://www.mdpi.com/1422-0067/21/3/774/s1.
