# Supplementary figures and images for: Transcriptome Analysis Reveals the Different Response to Toxic Stress in Rootstock Grafted and Non-Grafted Cucumber Seedlings
Source: Int J Mol Sci. 2020 Jan 24;21(3):774. doi: 10.3390/ijms21030774 (PMC7037640; doi:10.3390/ijms21030774)

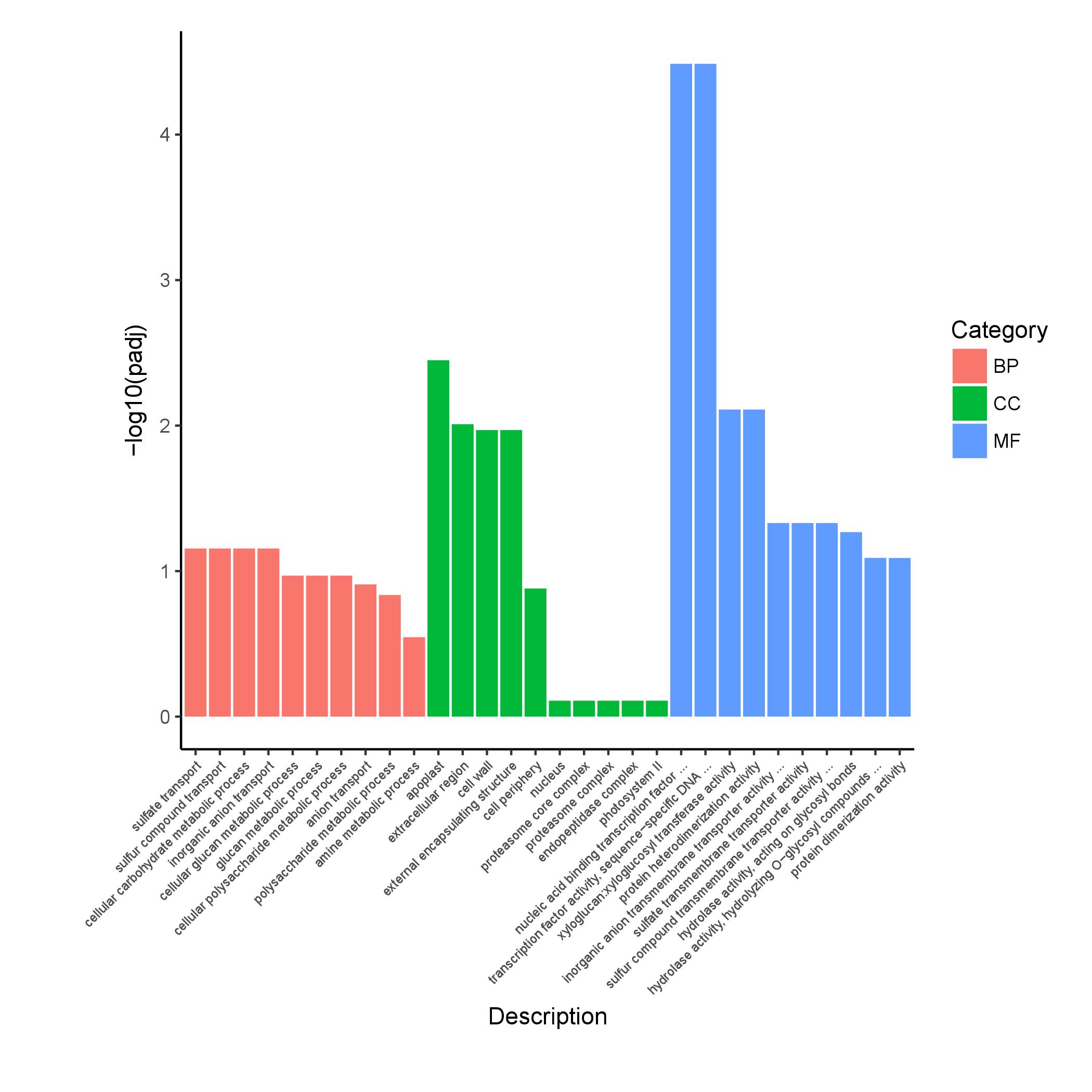

Supplement: Supplementary file 1 [file ijms-21-00774-s001.zip › supplementary files/Figure S1. RG_CKvsNG_CK_all_GObar.jpg]

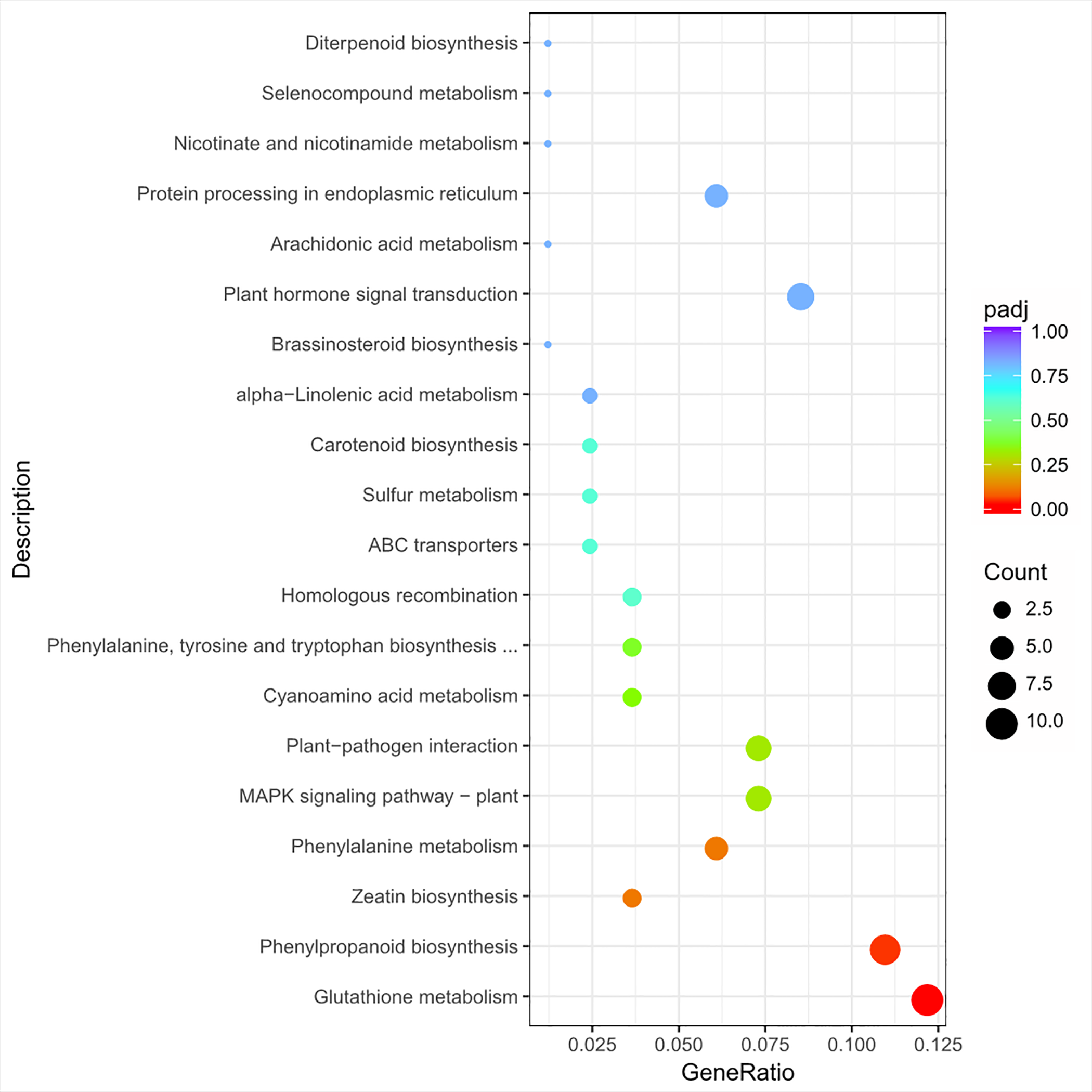

Supplement: Supplementary file 1 [file ijms-21-00774-s001.zip › supplementary files/Figure S2. RG_CKvsNG_CK_all.KEGGdot.tif]
